# Association between uremic toxin-anthranilic acid and fibrinolytic system activity in predialysis patients at different stages of chronic kidney disease

**DOI:** 10.1007/s11255-017-1729-1

**Published:** 2017-10-22

**Authors:** Tomasz W. Kaminski, Krystyna Pawlak, Malgorzata Karbowska, Michal Mysliwiec, Waldemar Grzegorzewski, Jakub Kuna, Dariusz Pawlak

**Affiliations:** 1 Department of Pharmacodynamics, Medical University of Bialystok, 2C Mickiewicza Str., 15-089 Białystok, Poland; 2 Department of Monitored Pharmacotherapy, Medical University of Bialystok, Białystok, Poland; 3 Department of Nephrology and Clinical Transplantation, Medical University of Bialystok, Białystok, Poland; 4 Department of Pharmacology and Toxicology, Faculty of Medical Sciences, University of Warmia and Mazury, Olsztyn, Poland

**Keywords:** Anthranilic acid, Fibrinolysis, Renal failure, Hemostasis, Fibrinolytic capacity

## Abstract

**Purpose:**

Chronic kidney disease (CKD) is an estimated risk factor for increased mortality and morbidity due to fibrinolytic system disturbances. Progressive loss of renal function leads to retention of uremic toxins. Anthranilic acid (AA) is a tryptophan-derived uremic toxin with multidirectional properties that can affect the hemostatic system. The goal of this study was to examine the association between AA and the parameters of fibrinolysis at different stages of CKD.

**Methods:**

Patients with CKD were divided into two groups: mild-to-moderate (*n* = 20) and severe-to-end-stage CKD (*n* = 28). Seventeen healthy volunteers served as an additional control group. Parameters of fibrinolysis, inflammation, and monocytes activation were determined by ELISA immune-enzymatic kits. AA levels were evaluated using high-performance liquid chromatography.

**Results:**

AA concentration and parameters of fibrinolysis: urokinase-type plasminogen activator (uPA), its soluble receptor (suPAR), tissue plasminogen activator (tPA), tissue plasminogen activator inhibitor-1 (PAI-1) and plasmin-antiplasmin complex (PAP) were significantly elevated in the CKD groups compared with the controls. The markers of inflammation, monocyte activation, and impaired kidney function were also increased in those with CKD. AA was positively correlated with the uPA/suPAR system in the early stages of CKD, whereas during severe-to-end-stage CKD, inverse relationships were observed between AA, tPA and PAI-1. Additionally, AA was an independent variable associated with tPA in patients with CKD overall and with uPA levels in the mild-to-moderate CKD group.

**Conclusions:**

Obtained results suggest for the first time the association between AA and the fibrinolytic system in CKD patients. The distinct relationship between AA and individual parameters of fibrinolysis appears to be dependent on CKD stage.

## Introduction

Chronic kidney disease (CKD) is an established risk factor for the occurrence of cardiovascular disease (CVD). Furthermore, CKD patients present with a significantly worsening quality of life and a 20-fold higher mortality rate compared with the overall population [1–3]. Patients with CKD suffer from dysfunctions in fibrinolytic activity, manifested by prothrombotic and bleeding tendencies. The exact etiology of the coexistence of these contrary hemostatic disorders in uremic conditions is poorly understood [4, 5]. Increased cardiovascular mortality and morbidity of patients with CKD is considered to be intensified due to increased concentrations of accumulated uremic toxins (UTs) [6, 7]

Defects in the activity of fibrinolysis are one of the most frequent disorders leading to an increased ratio of CVD events during CKD. The initial step that activates the fibrinolytic system is a conversion of plasminogen to plasmin through the influence of plasminogen activators: tissue-type (tPA) or urokinase-type (uPA) [8]. Because of immediate inhibition of released plasmin by α2-antiplasmin and the appearance of plasmin-antiplasmin complex (PAP), the measurement of PAP levels is considered to be an adequate assessment of *in vivo*-formed plasmin [9]. The main inhibitor of fibrinolytic activity is a plasminogen activator inhibitor-1 (PAI-1). The complementary factor of this system, which may regulate procoagulant-hyperfibrinolysis counterbalance, is soluble urokinase-type plasminogen activator receptor (suPAR)—a receptor for uPA in its soluble form [10].

Previous studies have shown enhanced l-tryptophan (Trp) degradation in patients with CKD [11, 12]. The primary degradation pathway of Trp is the kynurenine pathway (KP). The KP-related UTs exert multidirectional effects towards the body and are classified as a separate group of UTs—indole derivatives—including anthranilic acid (AA). AA is produced from kynurenine (Kyn) through kynureninase and the elevation of AA levels has been observed in disorders such as schizophrenia, rheumatoid arthritis, and diabetes [13]. Currently, no effective and efficient methods exist to remove accumulated AA from the body, which makes this toxin important for CKD patients in uremic conditions. Pawlak et al. [11] provided evidence of the connection between AA levels and endothelial adhesion molecules suggesting the possibility of triggering the adhesion of leukocytes to the vessel wall by Trp metabolites from KP. Furthermore, AA positively correlates with the levels of von Willebrand Factor (vWF), thrombomodulin (TM), and soluble endothelial adhesion molecules in patients undergoing hemodialysis [14]. These factors are the markers of endothelial dysfunctions and play a role in the maintenance of hemostasis. Our and other recent studies have shown that adhesion molecules, TM, and vWF are reliable predictors of CVD prevalence in CKD patients [15, 16]. In a renal-induced hypertension rat model, the plasma levels of AA were associated with increased blood pressure and seemed to be involved in CVD progression [17]. The renin–angiotensin–aldosterone system (RAAS) plays an important role in maintaining balance of the circulatory system during CKD and its activity is affected by elevated levels of Trp-derived uremic toxins [18, 19]. Moreover, the main metabolite of AA-3-hydroxyanthranilic acid has been found to be independently associated with monocyte chemoattractant protein-1 (CCL2) and macrophage inflammatory protein-1beta (CCL4) in CKD patients [20].

Aside from the above-mentioned observations, no further available studies exist regarding the role of AA towards the hemostatic system, especially fibrinolysis. We have previously reported that UTs that are metabolites of Trp, such as Kyn, kynurenic acid, and quinolinic acid, are associated with the vast majority of components of fibrinolysis. The possible existence of KP metabolites’ effects towards fibrinolytic activity and capacity has been suggested [11].

This background leads us to focus on the connections between AA concentrations and parameters of the fibrinolytic system in patients with chronic exposure to elevated levels of AA during CKD. The purpose of this study was to estimate whether or not AA is associated with the parameters of fibrinolysis and conditions related to decrement in renal function. We also assessed if AA is an independent variable for the evaluated parameters.

## Materials and methods

### CKD patients

In total, 48 clinically stable, non-dialysis patients on conservative treatment were enrolled in the study. The main inclusion criteria were: the classic symptoms of persistent CKD with respect to current guidelines, stability and full clinical monitoring of the patient, written consent, and willingness to cooperate with hospital personnel. The exclusion criteria were: receiving immunosuppressive treatment, non-steroidal anti-inflammatory drugs, antioxidants such as vitamin C or E, or allopurinol shortly before and during the study. Patients with active infections and autoimmune diseases were excluded from further analysis. The overall CKD group (A) was divided into two subgroups in view of CKD progression: subgroup B included mild-to-moderate CKD (II and III stages of CKD, *n* = 20), while subgroup C comprised severe-to-end-stage CKD (IV and V stages of CKD, *n* = 28). The criterion used for dividing the population into subgroups was the value of estimated glomerular filtration rate (eGFR) and was based on current guidelines. All parameters were assessed with regard to the overall CKD group and subgroups. eGFR was calculated according to the guidelines of The National Kidney Foundation. Body mass index was calculated by dividing weight in kilograms by height in meters squared.

### Control group

The control group comprised 17 sex- and age-matched healthy volunteers. None had received drugs or vitamins during the study. Moreover, all were on a standard diet and had no history of CKD, CVD, and diabetes mellitus.

### Ethical approval

The study protocol was approved in accordance with the ethical guidelines of the Local Ethical Committee of the Medical University of Bialystok (No. R-I-002/47/2017) and written consent was obtained from each study participant. The study was conducted according to the Declaration of Helsinki.

### Blood sampling and laboratory measurements

Blood samples were taken from the antecubital vein of CKD patients and healthy controls between 8 and 9 a.m. under fasting conditions with 3.8% sodium citrate at a proportion of 1:9 (v/v). Then, the collected blood was centrifuged at 3500 rpm for 20 min at 4 °C to obtain the plasma. All prepared samples were aliquoted and stored at – 80 °C until assayed.

Biochemical and hematological parameters were determined by standard laboratory methods.

### Determination of anthranilic acid

AA concentrations were determined according to Herve et al. [21]. The column effluent was monitored by using a programmable fluorescence detector. The optimized conditions were determined by recording fluorescence spectra with a stop-flow technique. Excitation and emission wavelengths were set at 320/420 nm for AA. The mobile phase comprised 50 mM acetic acid and 0.25 M zinc acetate (pH − 4.9), containing 1.2% of acetonitrile that was pumped at 0.2 ml/min. Chemical reagents were purchased from Sigma Aldrich (St. Louis, MO, USA) and Merck Co. (Kenilworth, NJ, USA).

### Parameters of the fibrinolytic system

The plasma levels of urokinase-type plasminogen activator (uPA), tissue plasminogen activator (tPA), soluble urokinase-type plasminogen activator receptor (suPAR), plasminogen activator inhibitor-1 (PAI-1) and plasmin-antiplasmin complex (PAP) were assayed using commercially available standard ELISA kits: (IMUBIND uPA, IMUBIND tPA ELISA, IMUBIND plasma PAI-1 ELISA, and IMUBIND suPAR ELISA from American Diagnostica Inc., Greenwich, CT, USA,; Plasmin-α-2-antiplasmin Complex from Technoclone GmbH, Wien, Austria).

### Markers of inflammation and monocytes activation

Plasma C-reactive protein levels were measured by high-sensitivity ELISA (Imuclone CRP (hs) ELISA, American Diagnostica Inc). Neopterin concentrations were also determined by ELISA kit (Demeditec Diagnostics GmbH, Kiel, Germany). All the ELISA assays were conducted accordingly to the instructions provided in manufacturers’ manuals under the same conditions.

### Statistical analysis

The normality of distribution was tested using the Shapiro–Wilk test and data were expressed as mean ± SD. The non-Gaussian data were presented as median (full range). The significant differences between groups were assessed by Student *t* test or nonparametric Mann–Whitney *U* test. The correlations between studied variables were determined by Spearman’s rank correlation analysis. Multiple regression analysis was performed using a stepwise model to determine the combined influence of variables on particular parameters of analyzed parameters. A two-tailed *P* value < 0.05 was considered to be statistically significant. Computations were performed using GraphPad 6 Prism software (GraphPad Software; La Jolla, CA, USA). The power of the analysis was estimated using StataIC 13 software (Stata Corp LLC, College Station, TX, USA).

## Results

Basal characteristics concerning biochemical and hematological parameters of CKD patients and healthy controls are given in Table 1. As expected, CKD patients had lower values of eGFR and higher concentrations of creatinine than controls. Moreover, similar remarks were observed between the two analyzed subgroups. Markers of inflammatory state (hs-CRP) and monocytes activation (neopterin) were more significantly elevated in the uremic group than in the controls; however, only the levels of neopterin were significantly changed between the subgroups. Moreover, CKD patients had significantly elevated levels of triglycerides and significantly decreased concentrations of albumin and total protein, whereas the concentrations of total cholesterol, bilirubin, and glucose remained unchanged. From hematological parameters, a statistically significant drop was observed in the values of red blood cells, hemoglobin, and hematocrit in CKD patients, whereas the amounts of white blood cells and platelets did not differ between the groups. Similar dependencies existed between the studied subgroups.

**Table 1:** Biochemical and clinical characteristics of control group and CKD patients

Parameter	Controls *n* = 17	CKD (group A) *n* = 48	Mild to moderate CKD (group B) *n* = 20	Severe to end-stage CKD (group C) *n* = 28
Sex (M/F)	7/10	19/29	8/12	11/17
Age (years)	47.7 ± 6.23	52.9 ± 15.7	50.5 ± 18	54.7 ± 15.9
BMI (kg/m^2^)	26.1 ± 3.37	23.9 ± 3.34	24.4 ± 3.11	23.6 ± 3.61
eGFR (mL/min/1.73 m^2^)	117 (105–125)	19.9 (5.6–127)***	58.8 (29.8–127)*_^^^_	12.4 (5.6–28.9)***^^^###^
Creatinine (mg/dL)	0.89 (0.29–1.18)	3.45 (0.78–7.83)***	1.2 (0.78–2.31)**^^^^^	5.03 (1.82–7.83)***^^^###^
hs-CRP (µg/ml)	1.15 (0.01–10.9)	2.98 (0.01–47)*	2.03 (0.01–45)	5.85 (0.02–47)**
Neopterin (nmol/L)	6.18 (0.4–12.91)	31.4 (5–110)***	28 (5–108)***	39.1 (5–110)***^#^
Glucose (mg/dL)	88 (67–114)	90 (45–155)	95.5 (76–155)	89 (45–121)^#^
Cholesterol (mg/dL)	193 (144–245)	198 (106–485)	194 (164–485)	200 (106–263)
Triglycerides (mg/dL)	67 (38–149)	148 (61–481)***	170 (61–620)***	147 (63–392)***
Total protein (g/dL)	6.41 ± 0.27	6.01 ± 0.94	5.78 ± 0.89**	6.21 ± 0.99
Albumin (g/dL)	4.41 ± 0.17	3.3 ± 1.18***	3.77 ± 1.13**	3.18 ± 1.27***^#^
Bilirubin (mg/dL)	0.34 ± 0.13	0.45 ± 0.2	0.43 ± 0.21	0.51 ± 0.21**
ALT (U/L)	33 (16–53)	27.5 (10–120)	28 (15–120)	25.5 (10–58)
Red blood cells (× 10^3^ µL)	4.55 ± 0.32	3.55 ± 0.68***	3.97 ± 0.77**^^^	3.31 ± 0.54***^###^
White blood cell (mg/dL)	5.76 ± 1.13	6.38 ± 2	6.93 ± 1.99*	5.99 ± 1.95
Platelets (× 10^3^ µL)	206 (132–312)	183 (76–376)	219 (115–359)	175 (76–376)
Hemoglobin (g/dL)	14.2 ± 1.32	11 ± 2.21***	12.2 ± 2.52*^^^	10.3 ± 1.82***^##^
Hematocrit (%)	42.2 ± 3.2	32.9 ± 5.99***	36.2 ± 6.81**^^^	31 ± 4.96***^##^

Data are shown as mean ± SD or median (range) depending on their normal or skewed distribution

*M* male, *F* female, *BMI* body mass index, *eGFR* estimated glomerular filtration rate, *hs*-*CRP* high sensitivity C-reactive protein, *ALT* alanine transaminase, *CKD* chronic kidney disease, *NS* non-significant

*,**,*** *P* values respectively < 0.05; < 0.01; < 0.001 (controls vs. others)

^^,^^,^^^^ *P* values < 0.05; < 0.01; < 0.001 (CKD vs. others)

^#,##,###^ *P* values < 0.05; < 0.01; < 0.001 (group B vs. group C)

### Additional baseline information

Patients received ACE-inhibitors (*n* = 24), Ca^2+^ blockers (*n* = 24), β-blockers (*n* = 20), α-blockers (*n* = 4), nitrates (*n* = 2) and angiotensin receptor blockers (ARBs) (*n* = 1). Polytherapy was prescribed in 28 cases. The causes of CKD included glomerulonephritis (*n* = 23), diabetic nephropathy (*n* = 8), polycystic kidney disease (*n* = 5), pyelonephritis (*n* = 3), hypertensive nephropathy (*n* = 2) and idiopathic nephropathy (*n* = 7). One patient was diagnosed with hepatitis C virus infection. CVD defined as the occurrence of myocardial infarction, angina pectoris, ischemic stroke, peripheral artery surgery, coronary revascularization procedures, and typical changes on coronary angiograms in the past were observed in 43.75% of patients.

### Levels of anthranilic acid

**Fig. 1:**
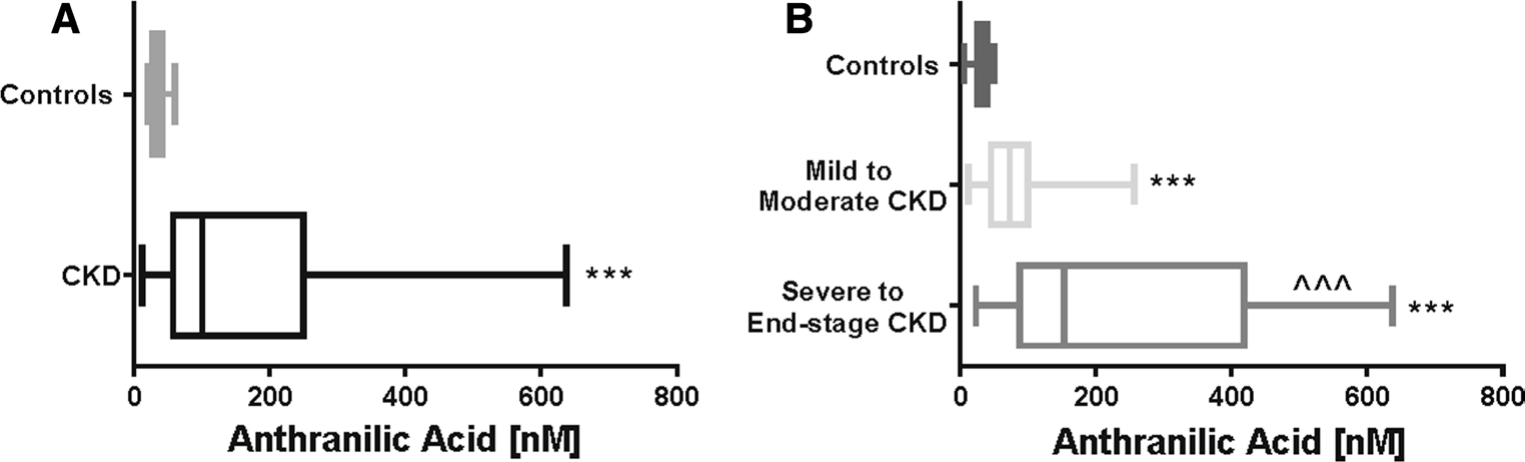
The levels of AA in control group and CKD group (**a**) and comparison of the levels of AA among the patients with the different stage of CKD (**b**). *,**,*** *P* values respectively < 0.05; < 0.01; < 0.001; (controls vs. others). ^^,^^,^^^^ *P* values respectively < 0.05; < 0.01; < 0.001; (severe to end-stage CKD vs. mild to moderate CKD). AA, anthranilic acid; CKD, chronic kidney disease

The concentrations of AA were more significantly elevated in CKD patients than in the control group (Fig. 1a). Furthermore, the concentrations of AA were significantly higher in patients with severe-to-end-stage CKD compared with patients with mild-to-moderate CKD (Fig. 1b).

### Changes in the parameters of the fibrinolytic system

**Fig. 2:**
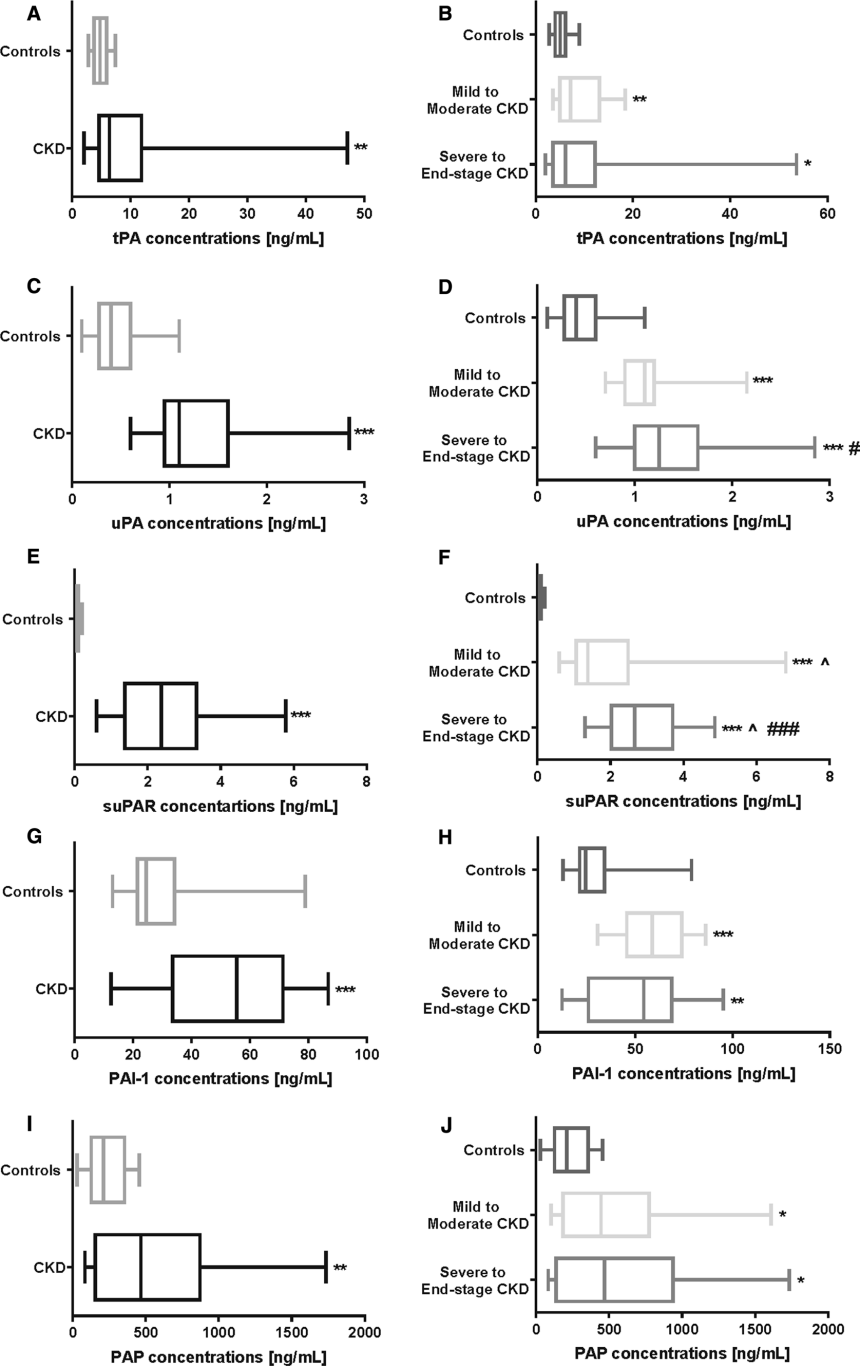
Parameters of the fibrinolytic system in healthy controls, overall CKD patients (**a**, **c**, **e**, **g**, **i**), and comparison of the fibrinolytic factors in the CKD subgroups (**b**, **d**, **f**, **h**, **j**). *,**,*** *P* values respectively < 0.05; < 0.01; < 0.001; (controls vs. others). ^^,^^,^^^^ *P* values respectively < 0.05; < 0.01; < 0.001; (CKD vs. others). ^#,##,###^ *P* values respectively < 0.05; < 0.01; < 0.001; (severe to end-stage CKD vs. mild to moderate CKD). tPA, tissue plasminogen activator; uPA, urokinase-type plasminogen activator; suPAR, soluble urokinase-type plasminogen activator receptor; PAI-1, plasminogen activator inhibitor-1; PAP, plasmin-α2-antiplasmin

All tested parameters of the fibrinolytic system (uPA, tPA, suPAR, PAI-1 and PAP) were significantly higher in the CKD patients compared with the controls (Fig. 2a). The measured fibrinolytic parameters, except for the suPAR concentrations, did not differ between the B and C subgroups (Fig. 2b).

### Correlations between anthranilic acid and fibrinolytic parameters

The concentrations of AA positively correlated with the overall levels of uPA and suPAR, whereas concentrations of tPA and PAI-1 correlated negatively with AA in patients with CKD overall (Table 2A). The only parameter of the fibrinolytic system that was not correlated directly with AA levels was PAP. Interestingly, the dependencies between AA and uPA as well as suPAR existed only in the mild-to-moderate CKD subgroup (Table 2B), whereas correlations with tPA and PAI-1 occurred only in the severe-to-end-stage CKD subgroup (Table 2C). None of above-mentioned correlations were observed in the control group. In addition, the levels of AA and uPA were independent variables associated with concentrations of tPA in the overall CKD group (Table 3). When multiple regression analysis was performed for each subgroup separately, AA was independently and positively associated with the levels of uPA in subgroup B (Table 4). Multiple regression in subgroup C did not show any statistically significant results. Neither cause of CKD nor pharmacotherapy influenced the measured parameters and obtained correlations.

**Table 2:** Correlations between the levels of AA and the parameters of fibrinolytic system in the overall CKD (A), mild to moderate CKD subgroup (B), and severe to end stage CKD subgroup (C)

Overall CKD (A)	*R*	*P* value
tPA	**−** **0.365**	**0.0115**
uPA	**0.345**	**0.0176**
suPAR	**0.470**	**0.0009**
PAI-1	**−** **0.393**	**0.0062**
PAP	0.0429	NS

Mild to moderate CKD (B)	*R*	*P* value
tPA	0.103	NS
uPA	**0.689**	**0.0008**
suPAR	**0.561**	**0.0125**
PAI-1	**−** 0.209	NS
PAP	0.152	NS

Severe to end-stage CKD (C)	*R*	*P* value
tPA	**−** **0.573**	**0.0018**
uPA	**−** 0.023	NS
suPAR	0.029	NS
PAI-1	**−** **0.458**	**0.0188**
PAP	0.0194	NS

Bold values indicate presence of statistically significant correlation

Results are shown as Spearman’s rank correlations coefficient (R) and its statistical significance (*P* value)

*AA* anthranilic acid, *tPA* tissue plasminogen activator, *uPA* urokinase-type plasminogen activator, *suPAR* soluble urokinase-type plasminogen activator receptor, *PAI*-*1* plasminogen activator inhibitor-1, *PAP* plasmin-α2-antiplasmin, *NS* non-significant

**Table 3:** Variables independently associated with tPA levels in the overall CKD group

Overall CKD	Independent variable	Regression coefficient	*P* value
tPA	AA	**−** 0.28	0.034
	uPA	0.32	0.036

Multiple *R* for variables in the model = 0.682, multiple *R* ^2^ = 0.466, adjusted *R* ^2^ = 0.388, *P* < 0.0015

Included variables: AA, fibrinolytic factors, renal insufficiency markers

*tPA* tissue plasminogen activator, *AA* anthranilic acid, *uPA* urokinase-type plasminogen activator

**Table 4:** Variables independently associated with uPA levels in the mild to moderate CKD subgroup

Mild to moderate CKD subgroup	Independent variable	Regression coefficient	*P* value
uPA*	AA	0.49	0.012

Multiple *R* for variables in the model = *0.899, multiple *R* ^2^ = *0.808, adjusted *R* ^2^ = *0.668, * *P* **<** 0.004

Included variables: AA, fibrinolytic factors, renal insufficiency markers

*AA* anthranilic acid, *uPA* urokinase-type plasminogen activator, *CKD* chronic kidney disease

### Correlations between analyzed parameters

As is shown in Table 5, the positive correlations were between the levels of AA and neopterin values. Moreover, reverse correlation between eGFR and AA levels occurred. No correlations were observed between the markers of inflammation and AA levels. In contrast, AA levels in the mild-to-moderate CKD subgroup correlated only with the levels of eGFR and neopterin (Table 5). In the severe-to-end-stage CKD subgroup, AA levels were associated with eGFR (Table 5).

**Table 5:** Correlations between levels of AA and factors related to decrement in renal function, inflammation, and monocytes activation

Factor correlated with AA	*R*/*P* value Overall CKD (A)	*R*/*P* value Mild to moderate CKD (B)	*R*/*P* value Severe to end-stage CKD (C)
eGFR	**−** **0.773**	**−** **0.564**	**−** **0.419**
	**<** **0.0001**	**0.0096**	**0.0296**
hsCRP	0.217	0.265	0.0398
	NS	NS	NS
Neopterin	**0.448**	**0.381**	0.093
	**0.0020**	**0.034**	NS

Bold values indicate presence of statistically significant correlation

Results are shown as Spearman’s rank correlations coefficient (R) and its statistical significance (*P* value)

*AA* anthranilic acid, *eGFR* estimated glomerular filtration rate, *NS* non-significant

## Discussion

CKD is a pandemic disease associated with an elevated level of UTs and increased risk for cardiovascular events. This study is the first to examine the association between AA and fibrinolytic components in patients with CKD on conservative treatment exposed to permanently elevated AA levels. Our research demonstrates the following: (1) AA and all analyzed parameters of the fibrinolytic system were elevated in patients with CKD compared with the controls; (2) AA concentrations were associated with levels of uPA and suPAR in the mild-to-moderate CKD subgroup as well as with PAI-1 and tPA in the severe-to-end-stage CKD subgroup; (3) AA levels are an independent variable for predicting tPA concentrations in the overall CKD group and for uPA levels in the mild-to-moderate CKD subgroup; (4) CKD patients are exposed to chronic inflammation and other conditions specific to progressive deterioration of kidney function, which may affect activation of both the fibrinolytic system and KP.

Our previous studies proved that KP is activated during CKD [11, 14, 17]. In this study, we observed a rise in AA concentrations. These data confirmed the activation of KP in the CKD group compared with the controls, which is in line with our previous and others’ observations [12, 20]. Moreover, the overall CKD group represents a statistically higher level of markers of inflammation, monocyte-to-macrophage transition and other parameters related to the conditions of chronic kidney function decline. Pro-inflammatory cytokine—interferon-ɤ (IFN-γ) is known to have the ability to induce the crucial enzyme of the KP, indoleamine 2,3-dioxygenase (IDO), and in this manner to accelerate production of Trp metabolites *via* the KP [22]. IDO activity can be induced by Th1-type cytokine IFN-γ in parallel to neopterin production in human monocyte-derived dendritic cells and macrophages [23], and this mechanism indirectly contributes to increased production of AA as well as other KP metabolites [24]. These assumptions lead to recognition of neopterin as a spinning mechanism activating the KP, which is strengthened by the positive correlation between AA levels and neopterin. However, data from our experiment did not show a correlation between the overall marker of the inflammatory state (hsCRP) and the plasma levels of AA in patients with CKD.

CKD patients are characterized by both impaired fibrinolytic capacity and hyperfibrinolysis leading to increased frequency of cardiovascular events [4]. Our research demonstrates the activation of the fibrinolytic system in renal failure’s conditions. This observation is in line with that of previous studies showing an increase in bleeding tendencies with simultaneous activation of counter-balance mechanisms required for maintaining proper hemostatic functions during CKD [11]. Importantly, we observed for the first time diversified relationships between AA levels and the main factors of the fibrinolytic system—a positive correlation was observed between AA and uPA/suPAR in the mild-to-moderate CKD subgroup, whereas an inverse relationship existed between AA and tPA/PAI-1 in the severe-to-end-stage CKD subgroup. This is an interesting observation because both uPA/suPAR and tPA/PAI-1 concentrations were higher in the CKD patients than in the healthy controls. In the mild-to-moderate CKD subgroup, the levels of AA were strongly correlated with both uPA/suPAR as well as with neopterin. Moreover, AA independently affected uPA levels. On the basis of the observed strong connection between uPA and suPAR, which directly affected PAP, we hypothesize that KP activation resulting in AA production participates in the activation of the fibrinolytic system in the early stages of CKD. In the severe-to-end-stage CKD subgroup, the levels of AA were inversely correlated with the main factors regulating the crucial steps of the fibrinolytic system—tPA and its inhibitor PAI-1. Moreover, AA and uPA independently affected tPA levels in the overall CKD group. These data suggest that AA may play contradictory roles in modulating the fibrinolytic system.

In mild CKD, AA possibly stimulates activity of fibrinolysis in a uPA/suPAR-dependent manner, whereas during the progression of CKD, the role of AA is changed, and this metabolite of KP may become an inhibitor of the fibrinolytic system. This suggests the unrevealed mechanism of AA action toward tPA and uPA synthesis, activity, or stability.

The results of this study indicate that many CKD-dependent factors, such as altering the KP, inflammation or monocyte activation, can partially interplay with measured parameters and associations due to the existence of multidimensional connections between these factors [25]. Moreover, uPA/suPAR and tPA/PAI-1 systems differ substantially in the mechanism of action and physiological significance. tPA is mainly involved in intravascular thrombolysis, whereas uPA exerts its activity through suPAR, which is presented in various types of cells [26]. Other studies show that tPA is implicated in fibrin degradation within the vasculature, while uPA causes lysis of the fibrin deposited at extravascular sites [27]. Impaired renal function leads to decreased clearance of uPA/suPAR, simultaneous increased systemic production, and the release of these compounds, resulting in accelerated conversion of plasminogen into plasmin [9, 27]. Although the direct association between AA and fibrinolytic activity, reflected by PAP levels, was not observed in this study, AA was directly correlated with suPAR, which in turn was the only fibrinolytic parameter associated with PAP levels. A recent study indicated that activity of uPA/suPAR complexes determinates the overall activity of the fibrinolytic system [28, 29], and our results are in line with these data. Our study confirms a previously observed increase in the levels of PAI-1 and shows an inverse correlation between AA and the parameter in the severe-to-end-stage CKD group. Hyper-expression of PAI-1 in renal tissue is one of the pleiotropic molecular mechanisms associated with RAAS hyperactivity and CKD progression. Furthermore, RAAS is ubiquitously expressed throughout the cardiovascular system and the kidney. Moreover, an interplay between the RAAS and the coagulation/fibrinolytic systems has been demonstrated in uremic conditions [30]. Hence, RAAS may be affected by elevated levels of AA, suggesting the existence of a previously unknown action of AA towards the system regulating blood pressure, extracellular volume, and vascular tone. Recent studies have shown that RAAS activity is modified by KP metabolites and other Trp metabolites such as indoxyl sulfate [17, 30]. Interestingly, introducing a ramipril-angiotensin-converting enzyme inhibitor reduced proteinuria and may be beneficial in reducing CKD-related mortality [31]. Moreover, our results suggest for the first time the indirect enhancement of fibrinolytic activity by AA that may be dependent on the progression of CKD, because the above-mentioned relationships did not exist in the control group.

Our study was observational, not mechanistic, in nature and was limited due to its cross-sectional design. However, for the first time, we investigated the relationship between concentrations of Trp-derived uremic toxin (AA), and parameters of fibrinolysis in predialysis patients with CKD. This allowed us to focus on chronic elevated levels of AA without the influence of hemodialysis that *per se* may affect hemostatic parameters [32]. Due to the relatively small number of patients, further prospective cohort studies are required. Further, subgroup allocation lowers the statistical power of the used tests. Additionally, we were unable to establish any mechanisms underlying the observed associations in view of various factors related to characteristic CKD conditions. Additional experimental support is needed to resolve several crucial issues in order to fully understand observed phenomena.

To conclude, our research has shown for the first time the association between the level of AA and the parameters of the fibrinolytic system in a group of CKD patients on conservative treatment. The distinct relationship between AA and individual parameters of the fibrinolytic system appear to be dependent on CKD stage. Obtained data suggest the existence of previously unknown properties of AA towards the fibrinolytic disturbances in this population. Although at this stage our results are only speculative, we can assume that AA may play a part in the various mechanisms involved in altering the fibrinolytic system during CKD.
